# Generalism and Specialism

**Published:** 1917-10-06

**Authors:** 


### The Hospital

The Workers' Newspaper of Administrative Medicine and Institutional Life, Administration, National Insurance and Health.

No. 1635, Vol. LXIII. SATURDAY, OCTOBER 6, 1917. PRICE ONE PENNY

### GENERALISM AND SPECIALISM.

"Specialism" and "specialist" are not very happy terms in relation to the practice of medicine. But they have a fairly firm hold, and the omens are that they have come to stay. Apart from their intrinsic defects, it has to be remembered that they enjoy no legal protection and are as much at the disposal of the advertising quack as they are within the right of the registered and qualified practitioner. Then they compel the admission of corresponding antonyms, and the horrors of generalism and generalist appear on the scene. Yet some terms must be found for existing facts and distinctions, and so far no voice has offered a more pleasing inspiration. It seems as if we must take the words as they stand, and strive to suffer them with what philosophy may be at our disposal. But we should welcome a less harsh alternative.

The things which the words signify are real enough, and with them a reckoning must be held. Medicine hears of them daily and on every hand. In part they have been created by the public, but there is also a large endorsement by the profession. If some repudiate them, others welcome and adopt them. It is the bare fact that of recent years, and to a growing extent, numbers of practitioners have restricted their activities to particular branches of medical and surgical work, and that these, for want of a better word, have been dubbed "specialists." The action has certainly its values, and the name is almost a necessary outcome from it. At all events, the thing is here, and so is the name. Even more significant is the frequency with which such a policy is proclaimed as the one and only true path of medical progress. We are told that no place exists any longer for the practitioner who aspires to an all-round equipment, and that the patient of the early future is to find his salvation in a journey from specialist to specialist until the whole of his anatomy has been inspected and valued by an expert eye. One practitioner one organ, or even one practitioner one disease—these are the mottoes which medicine is recommended to inscribe over its portals.

Now we are by no means anxious to affirm that this line of argument is completely free from truth and value. On the contrary, we welcome the contention that the expert who cultivates a restricted outlook has a place, and an important place, both in medical treatment and in the extension of medical knowledge. But that he monopolises the whole of these fields we do not agree. He may render, and does render, essential service both in the one and the other, but this is not to say that there is no need for another voice. The current doctrine contains a truth, but it is not the whole truth, and the emphasis which is often lent to it is apt to be misleading. There is something to be said on the other side of the question, and both the reputation of medicine and the public interest demand that the whole position should be kept in view.

One opportunity there is which is most certainly reserved for the highly trained expert. It is found in all those departments where skill of craftsmanship is necessary. Operations and manipulations which demand delicacy and dispatch can only be successfully undertaken by those who are constantly engaged in their performance. The achievement is comparable to a skilled performance on a musical instrument. Both in the one and in the other the nervous and muscular organisations must not only be trained to a certain level of efficiency, but they must be kept at this level by constant practice. The extraction of a cataract, the removal of a tumour from the vocal cords, for example, are pieces of craftsmanship which are possible only to those whose dexterities of brain and hand are cultivated and maintained by daily discipline. Here then the expert or specialist enjoys an undisputed field, and to him we must look for advances and improvements in this field as well as for individual performances. Again, let it be allowed that the medical world is so extended and is so detailed that no man may be authoritative or efficient in all departments. Hence there will be occasions in reference both to diagnosis and to treatment when the position will be beyond the competence of the practitioner who has not a special personal experience in the question immediately at issue; and even in the complications and varieties of what may be called ordinary cases there is often room for the help of senior and experienced judgment. Here again, therefore, the expert or specialist may from his peculiar knowledge and training find an opportunity and a claim. On a particular and given point he speaks with the word of authority, and his help may be most valuable and welcome.

Yet, even when all these considerations have their due weight, there is still the commending fact that in each individual instance the welfare of a patient is at stake. And a patient is not a mere collection of organs on each one of which an expert may pass a judgment in accordance with a more or less accepted standard. There is a man or woman or child in issue, and if wise direction is to be afforded some one who is competent to develop an intelligent survey of all relevant considerations must stand in a position of authority. What the expert or specialist advises about the particular organ which is his proper field is, of course, important. But, after all, the patient remains the supreme question, and knowledge of the patient is no part of the specialist's programme. This is the other side of the picture, and it needs attention when so many voices exalt the specialist and say that there is room for none other. In our judgment there is need to insist that such a doctrine is misleading and that, while seeking specially expert help when this is needed, the practitioner who cultivates a wide outlook on his profession and trains himself on this basis is, and will remain, a valuable and necessary exponent of the medical art.

